# Migration of atrial septal occluder device to the thoracic aorta: case report

**DOI:** 10.1590/1677-5449.202401062

**Published:** 2025-07-28

**Authors:** Felipe Carrasco Ferreira Dionisio, Pedro Henrique Amaral Ângelo da Silva, Bruno Lima Moreira, Augusto Kreling Medeiros

**Affiliations:** 1 Associação Beneficente Síria, Hospital do Coração – HCor, São Paulo, SP, Brasil.; 2 Hospital DF Star Rede D’Or, Brasília, DF, Brasil.; 3 Hospital Beneficência Portuguesa de São Paulo, São Paulo, SP, Brasil.

**Keywords:** radiology, vascular surgical procedures, atrial septal defect, prostheses and implants, aorta, thorax

## Abstract

Atrial septal defect (ASD) is a common cardiac defect with significant implications if left untreated. Although open heart surgery is the traditional approach, transcatheter closure devices, such as the Amplatzer™ Septal Occluder Device, have gained prominence due to advantages like shorter hospital stays and reduced costs. Among potential complications, device migration is a rare complication, with an incidence of 0.5 to 1.1%.

We report a rare case of migration of an Amplatzer™ device in an asymptomatic patient, diagnosed 6 months after its implantation. After detecting the issue, the medical team opted for percutaneous device removal followed by open surgery to correct the ASD.

In conclusion, managing Amplatzer™ device embolization requires careful consideration of the patient’s circumstances and device anatomy. This case highlights the importance of correlating clinical and imaging findings when selecting the management approach and assessing the feasibility of a less invasive approach in cases of late migration.

## INTRODUCTION

Atrial septal defect (ASD) is one of the most common congenital cardiac defects. Some cases are identified early in childhood, while others remain undetected until adulthood, becoming hemodynamically significant over time. If left uncorrected, they can cause right ventricular overload, leading to heart failure, increased pulmonary vascular resistance, emboli, and atrial arrhythmia.^1,2^

While open surgery is a well-established method for repairing septal defects, improvements in devices for transcatheter closure have made this technique increasingly common. Studies suggest that these devices offer comparable efficacy to open surgery, with shorter hospital stays and reduced costs.^3^

In this context, Amplatzer™ septal occluder devices are widely used for minimally invasive closure. However, they are not entirely free from complications, among which migration is a rare event, with incidence ranging from 0.5 to 1.1% of cases.^4^

In this article, we report a rare case of migration of an Amplatzer™ device to the proximal thoracic descending aorta that was detected in the sixth month following implantation during a routine Doppler ultrasonography in an asymptomatic patient. This case report illustrates a rare complication during the postoperative period after placement of an atrial septal occlusion device and discusses the corresponding imaging findings and the treatment options available.

The protocol was approved by the Ethics Committee at our institution (CAAE – 75966923.4.0000.5483 and decision number 6.614.692).

## CASE DESCRIPTION

An asymptomatic 42-year-old man presented at our service with a referral letter recommended urgent admission having undergone an echocardiogram at an external provider for 6-month follow-up of percutaneous placement of an Amplatzer™ cardiac device to correct an *ostium secundum* ASD. It had not been possible to locate the device in its expected topography during the examination, suggesting the possibility of migration/embolization. The patient’s reports suggested that the examinations performed immediately postoperatively and for control at 3 months after the procedure had been normal.

During the physical examination at admission, the patient was free from acute discomfort, blood pressure was 173 / 80 mmHg, there was no fever or signs of cyanosis (oxygen saturation of 96% in room air), heart rate and rhythm were normal, and there was no evidence of heart murmur.

**Figure 1:**
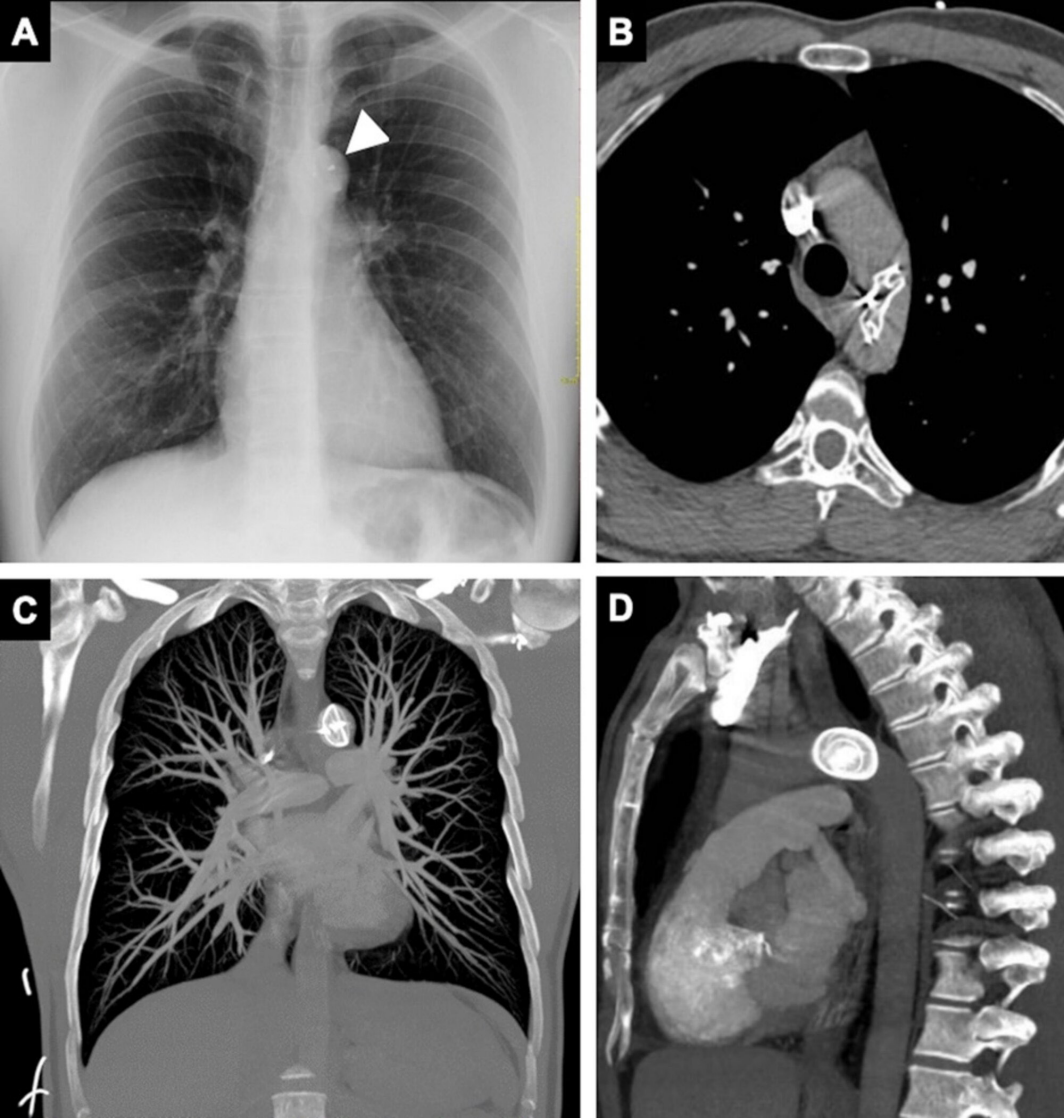
(A) Chest X-ray (front view) showing the anomalous position of the septal occluder device in the proximal descending thoracic aorta projection (arrowhead); (B-D) Computed tomography with iodinated contrast confirmed the position of the device in the proximal descending thoracic aorta and ruled out complications, such as pulmonary thromboembolism or aortic thrombosis.

He was admitted to hospital by the cardiology team. A chest X-ray revealed dense material with a circular shape in the region of the proximal descending thoracic aorta, compatible with the cardiac device fitted previously (Figure 1A). No devices were identified in the cardiac projection.

**Figure 2:**
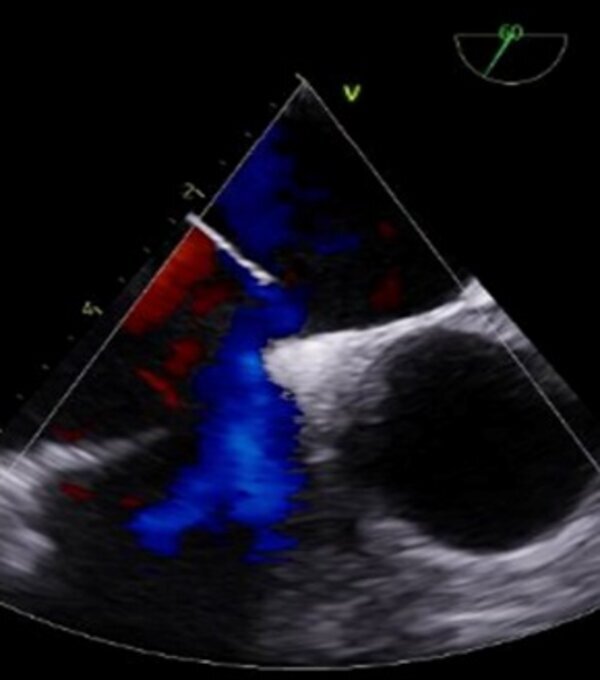
Transesophageal echocardiogram revealed persistence of an *ostium secundum* interatrial communication with a diameter of 7.5 mm, causing left-right shunt.

**Figure 3:**
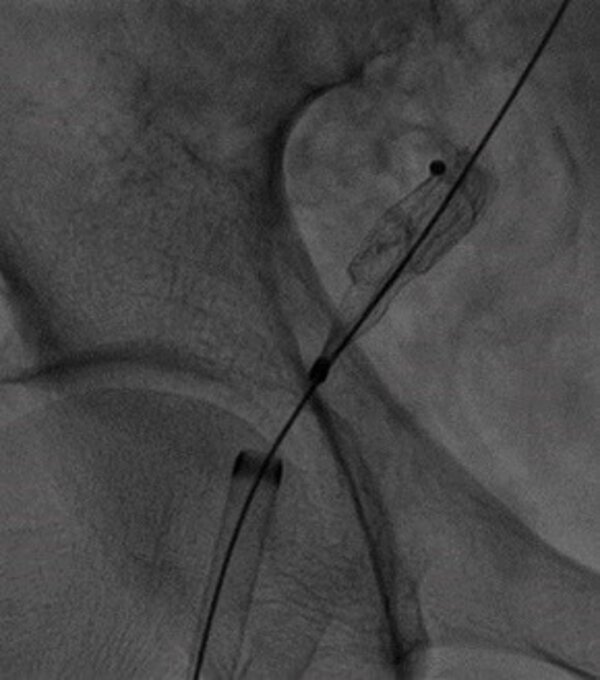
Fluoroscopic image of percutaneous removal of the device via the right iliofemoral arterial axis.

A subsequent angiotomography confirmed the Amplatzer™ device in an anomalous position in the proximal descending thoracic aorta and helped to rule out other complications, such as thromboembolism or formation of thrombi adjacent to the device (Figure 1B-D). Once the device had been precisely located, the patient underwent transesophageal Doppler ultrasonography, which showed that the interatrial communication with left-right shunt was still present (Figure 2). After analysis of the images and discussion of the case, the interventional cardiology team decided to attempt percutaneous removal of the device, via the right iliofemoral arterial axis (Figure 3) after puncture of the right femoral artery by the Seldinger technique. Aortography was used to locate the prosthesis and a 12F radiopaque sheath was inserted, followed by a snare catheter, which was advanced up to the proximal descending thoracic aorta, with capture and displacement of the migrated device. The device was found anchored in the femoral artery, requiring a longitudinal incision in this artery to enable removal of the device under direct visualization. After removal of the device, angiotomography was performed again, with no further significant findings, ruling out potential complications related to embolization of the Amplatzer™ device, such as thrombi or other vascular injuries (lesions). Approximately 2 weeks later, the patient underwent atrial septoplasty via a median sternotomy, with closure of the ASD using bovine pericardium, with no postoperative complications.

## DISCUSSION

Modern cardiac devices have both temporary and permanent applications and are used to treat a range of cardiac conditions. These devices include those for ASD occlusion.^1,5^ Several different studies have demonstrated that the percutaneous technique for ASD correction is as safe and effective as open surgery, with similar results. Advantages of percutaneous occlusion include no need for extracorporeal circulation, reduced postoperative discomfort, and shorter hospital stay.^3^

In 1997, Kurt Amplatz developed a self-expandable device made from nitinol wires (a nickel and titanium alloy) comprising two circular discs connected by a short central waist. This device became known as the Amplatzer™ septal occluder and was approved by the Food and Drug Administration (FDA) in 2001.^6^

In addition to its use for closure of ASDs, the Amplatzer™ device is also used to treat other types of heart defects, such as patent foramen ovale and ventricular septal defect. A range of different models and sizes are available, enabling choice of the most appropriate device tailored to each patient, considering the size and location of the defect.

The overall rate of complications after the percutaneous ASD closure procedure varies from 6.1% to 11.1%. The most common complications are embolization and malpositioning.^2^

A device that becomes displaced can migrate to several different areas, including the following: main pulmonary artery, left ventricle, left atrium, thoracic ascending aorta, aortic arch, or thoracic descending aorta. In the majority of cases, the device migrates to the main pulmonary artery, as shown by a study by Chessa et al.,^7^ in which migration to this artery occurred in 89% of cases analyzed.^7^ Migration is most often detected during the first 24 hours after the procedure, underscoring the importance of conducting Doppler ultrasonography immediately or during the first few hours after fitting the device.^2,8^ In this scenario, Doppler ultrasonography plays a crucial role after placement of an Amplatzer™ device, enabling determination of its exact position and detection of residual shunts. In contrast, cases of embolization to the aortic arch or to the thoracic descending aorta in the late postoperative period are extremely rare.^2^ In specific situations, primarily later cases, computed tomography is a valuable tool for evaluating devices, especially in relation to embolizations. It enables the precise location of the Amplatzer™ and its anatomic relationships to be determined, making it essential for management and choice of removal technique. Moreover, computed tomography facilitates assessment of device migration or protrusion into anatomic structures that might not be visualized with confidence using transthoracic echocardiogram.

With regard to management of cases of Amplatzer™ device embolization, studies indicate that percutaneous removal is a well-established option when embolization occurs soon after fitting the device. However, in late embolization cases, in which there is a high probability of endothelization of the prosthesis, removal by open surgery may be safer, because of the increased risk of injury to the vascular wall.^4^ Taking into account the risk-benefit balance in asymptomatic patients, conservative management may be considered (observation, without immediate removal of the device).^4,8^ In our case, the patient was asymptomatic and, after analysis of the images, ruling out complications adjacent to the device, such as formation of thrombi or endothelization, the medical team chose percutaneous removal of the device, using the right femoral artery.

In summary, management of Amplatzer™ device embolization must consider several factors, including the time of migration, the probability of endothelization, the clinical condition of the patient, and the anatomy of the device. Assessment with different imaging methods and their correlation plays a fundamental role in choice of the best management approach. In our case, correlation of clinical findings with the imaging exams was crucial, since it enabled us to start with the less invasive treatment, with intraoperative confirmation that the migrated device was free from vascular adhesion and percutaneous removal of the device via the right iliofemoral arterial axis.
