# Supplementary material for: Migration of atrial septal occluder device to the thoracic aorta: case report
Source: J Vasc Bras. 2025 Jul 28;24:e20240106. doi: 10.1590/1677-5449.202401062 (PMC12315574; doi:10.1590/1677-5449.202401062)
Supplement: Translation

# Translation: Migração de dispositivo de oclusão do septo interatrial para a aorta torácica: relato de caso

**DOI:** 10.1590/1677-5449.202401061

**Authors:** Felipe Carrasco Ferreira Dionisio, Pedro Henrique Amaral Ângelo da Silva, Bruno Lima Moreira, Augusto Kreling Medeiros

**Affiliations:** 1 Associação Beneficente Síria, Hospital do Coração – HCor, São Paulo, SP, Brasil.; 2 Hospital DF Star Rede D’Or, Brasília, DF, Brasil.; 3 Hospital Beneficência Portuguesa de São Paulo, São Paulo, SP, Brasil.

**Keywords:** radiologia, procedimentos cirúrgicos vasculares, defeito do septo atrial, próteses e implantes, aorta, tórax

## Abstract

O defeito do septo atrial (DSA) é um defeito cardíaco comum, com implicações significativas se não tratado. Embora a cirurgia aberta seja uma abordagem tradicional, os dispositivos de fechamento transcateter, como os dispositivos oclusores septais Amplatzer™, têm ganhado destaque devido às vantagens, como menor tempo de internação e custos reduzidos. Dentre suas possíveis complicações, a migração do dispositivo é uma complicação rara, com uma incidência de 0,5 a 1,1%.

Relatamos um caso raro de migração de um dispositivo Amplatzer™ em um paciente assintomático, diagnosticado no sexto mês posterior à sua implantação. Após a detecção do problema, a equipe médica optou pela remoção percutânea do dispositivo, seguida de cirurgia aberta para corrigir o DSA. A avaliação por métodos de imagem desempenhou um papel crucial na determinação do plano de tratamento.

Em suma, o manejo de embolização de dispositivos Amplatzer™ requer consideração cuidadosa das circunstâncias do paciente e da anatomia do dispositivo. Este caso destaca a importância da correlação clínica e de imagem na escolha da abordagem terapêutica e a viabilidade de uma abordagem menos invasiva em casos de migração tardia.

## INTRODUÇÃO

O defeito do septo atrial (DSA) é um dos defeitos cardíacos congênitos mais comuns, sendo que alguns são identificados precocemente na infância, enquanto outros permanecem ocultos até a vida adulta, tornando-se hemodinamicamente significativos com o tempo. Quando não corrigidos, podem causar sobrecarga no ventrículo direito, levando a insuficiência cardíaca, aumento da resistência vascular pulmonar, embolias e arritmias atriais^1,2^.

Embora a cirurgia aberta seja uma abordagem bem estabelecida para corrigir esses defeitos septais, o avanço dos dispositivos de fechamento transcateter tornou esta técnica cada vez mais comum. Estudos sugerem que esses dispositivos oferecem eficácia comparável à da cirurgia aberta, com menor tempo de internação e custos reduzidos^3^.

Nesse cenário, os dispositivos oclusores septais Amplatzer™ são amplamente utilizados para o fechamento minimamente invasivo. No entanto, não estão isentos de complicações, sendo a migração um evento raro, o qual, especificamente, ocorre em cerca de 0,5 a 1,1% dos casos^4^.

Neste artigo, relatamos um caso incomum de migração de um dispositivo Amplatzer™ para a aorta torácica descendente proximal, detectada no sexto mês posterior à implantação após realização de eco-Doppler de rotina em um paciente assintomático. Este caso ilustra uma complicação rara no pós-operatório de dispositivos de oclusão septal atrial e discute os achados de imagem correspondentes, bem como as opções terapêuticas disponíveis.

O protocolo foi aprovado pelo Comitê de Ética de nossa instituição (CAAE - 75966923.4.0000.5483 e parecer 6.614.692).

## DESCRIÇÃO DO CASO

Um homem de 42 anos, assintomático, procurou o serviço de emergência com uma carta de internação de urgência após a realização de um ecocardiograma em serviço externo, o qual foi realizado de rotina para seguimento após 6 meses de implantação percutânea de dispositivo cardíaco Amplatzer™ para correção de DSA do tipo *ostium secundum*. Nesse exame, não foi possível identificar o dispositivo na sua topografia habitual, inferindo sua possível migração/embolização. Relatos do paciente inferiam normalidade dos exames realizados no pós-operatório imediato e em controle após 3 meses do procedimento.

Durante o exame físico de admissão, o paciente estava sem desconforto agudo, apresentando uma pressão arterial de 173 / 80 mmHg, afebril, sem sinais de cianose (saturação de oxigênio de 96% em ar ambiente), frequência e ritmo cardíacos normais, e não havia evidência de sopro cardíaco.

Foi solicitada a internação hospitalar sob os cuidados da equipe de cardiologia. Uma radiografia de tórax foi realizada, revelando a presença de material denso em formato circular na região da aorta torácica descendente proximal, compatível com o dispositivo cardíaco previamente implantado (Figura 1A). Não foram identificados dispositivos na projeção do coração.

**Figura 1:** (A) Radiografia de tórax (vista frontal) mostra a posição anômala do dispositivo oclusor septal na projeção da aorta torácica descendente proximal (cabeça de seta); (B-D) Tomografia computadorizada com contraste iodado confirmou a posição do dispositivo na aorta torácica descendente proximal e descartou complicações, como tromboembolismo pulmonar ou trombose aórtica.

Uma angiotomografia subsequente confirmou a presença do Amplatzer™ em uma posição anômala na aorta torácica descendente proximal e ajudou a descartar outras complicações, como tromboembolismo ou formação de trombos adjacentes ao dispositivo (Figura 1B-D). Após a localização precisa do dispositivo, o paciente foi submetido a um eco-Doppler transesofágico, que identificou persistência da comunicação interatrial com *shunt* da esquerda para a direita (Figura 2). Realizada análise das imagens e discussão do caso, a equipe de cardiologia intervencionista optou pela remoção percutânea do dispositivo, a qual foi realizada pelo eixo arterial iliofemoral direito (Figura 3) através de punção da artéria femoral direita pela técnica de Seldinger, sendo realizada aortografia para localização da prótese e introduzida bainha radiopaca tamanho 12 Fr e cateter laço até a aorta torácica descendente proximal, promovendo a captura e o deslocamento do dispositivo migrado, o qual apresentou-se ancorado na artéria femoral, sendo necessária incisão longitudinal dessa artéria e retirada por visão direta. Posteriormente à remoção do dispositivo, uma nova angiotomografia foi realizada, sem mais achados significativos, descartando potenciais complicações relacionadas à embolização do Amplatzer™, como trombos ou outras lesões vasculares. Aproximadamente 2 semanas depois, o paciente foi submetido a atriosseptoplastia por meio de esternotomia mediana e fechamento do DSA com pericárdio bovino, evoluindo sem complicações pós-operatórias.

**Figura 2:** Ecocardiograma transesofágico revelou a persistência de uma comunicação interatrial do tipo *ostium secundum* com 7,5 mm de diâmetro, resultando em um shunt da esquerda para a direita.

**Figura 3:** Imagem fluoroscópica da retirada percutânea do dispositivo através do eixo arterial iliofemoral direito.

## DISCUSSÃO

Dispositivos cardíacos modernos têm aplicações tanto temporárias quanto permanentes e são utilizados no tratamento de diversas condições cardíacas. Entre esses dispositivos, estão os de oclusão do DSA^1,5^. Vários estudos demonstraram que a técnica percutânea para correção de DSAs é tão segura e eficaz quanto a cirurgia aberta, com resultados semelhantes. Entre as vantagens relatadas da oclusão por via percutânea, estão a não necessidade de circulação extracorpórea, menor desconforto pós-operatório e tempo de internação reduzido^3^.

Em 1997, Kurt Amplatz desenvolveu um equipamento autoexpansível feito de uma malha de fios de nitinol (uma liga de níquel e titânio) com dois discos redondos e uma conexão curta entre eles. Esse dispositivo ficou conhecido como oclusor septal Amplatzer™ e foi aprovado pela Food and Drug Administration (FDA) em 2001^6^.

Além de ser utilizado para o fechamento de DSAs, o Amplatzer™ também é empregado para tratar outros tipos de defeitos cardíacos, como o forame oval patente e a comunicação interventricular. Existem diferentes modelos e tamanhos de dispositivos disponíveis, permitindo uma escolha mais adequada e personalizada para cada paciente, levando em consideração o tamanho e a localização do defeito.

A taxa de complicações gerais após o procedimento de fechamento percutâneo de DSA varia de 6,1% a 11,1%. Entre essas complicações, a embolização e o posicionamento inadequado são as mais frequentes^2^.

Um dispositivo deslocado pode migrar para diferentes estruturas anatômicas, como a artéria pulmonar principal, ventrículo esquerdo, átrio esquerdo, aorta torácica ascendente, arco aórtico ou aorta torácica descendente. Na maioria dos casos, o dispositivo é direcionado para a artéria pulmonar principal, como evidenciado no estudo realizado por Chessa et al.^7^, em que a migração para essa artéria ocorreu em 89% dos casos avaliados. A migração é mais frequentemente detectada nas primeiras 24 horas após o procedimento, ressaltando a importância de realizar um eco-Doppler imediatamente ou nas primeiras horas após a implantação do dispositivo^2,8^. Nesse contexto, o eco-Doppler desempenha um papel crucial após a colocação do dispositivo Amplatzer™, permitindo verificar seu posicionamento exato e detectar shunts residuais. Por outro lado, a embolização para o arco aórtico e para a aorta torácica descendente é extremamente rara em casos de pós-operatório tardio^2^. Em situações específicas, principalmente em casos mais tardios, a tomografia computadorizada é uma ferramenta valiosa para avaliar os dispositivos, especialmente em relação às embolizações. Ela permite uma caracterização precisa da localização do Amplatzer™ e de suas relações anatômicas, sendo fundamental para o manejo e a escolha da técnica de retirada. Além disso, a tomografia computadorizada auxilia na avaliação de protrusões e/ou migrações de dispositivos para estruturas anatômicas que poderiam não ser visualizadas com confiança através de ecocardiograma transtorácico.

Quanto ao manejo de casos de embolização do Amplatzer™, estudos indicam que a retirada percutânea é uma opção bem estabelecida quando a embolização ocorre logo após a implementação do dispositivo. No entanto, em casos de embolização tardia, nos quais a probabilidade de endotelização da prótese é alta, a retirada por cirurgia aberta pode ser mais segura, devido ao maior risco de lesão na parede vascular^4^. Levando em conta o risco-benefício em pacientes assintomáticos, pode-se optar pela conduta conservadora (observação, sem remoção do dispositivo a princípio)^4,8^. Em nosso caso, o paciente estava assintomático e, após a análise das imagens, que afastaram complicações adjacentes ao dispositivo, como formação de trombos ou endotelização, a equipe médica optou pela remoção percutânea do dispositivo, utilizando a artéria femoral direita.

Em síntese, o manejo de embolização de dispositivos Amplatzer™ deve levar em consideração vários fatores, incluindo o momento da migração, a probabilidade de endotelização, a condição clínica do paciente e a anatomia do dispositivo. A avaliação por diferentes métodos de imagem e sua correlação desempenham um papel fundamental na determinação da melhor abordagem. Em nosso caso, a correlação clínica com os exames de imagem foi crucial, pois permitiu indicar inicialmente o tratamento menos invasivo, que durante a sua realização confirmou a não aderência vascular do dispositivo migrado, sendo realizada a remoção percutânea do dispositivo via eixo arterial iliofemoral direito.
